# The Molecular Anatomy of Spontaneous Germline Mutations in Human Testes

**DOI:** 10.1371/journal.pbio.0050224

**Published:** 2007-08-28

**Authors:** Jian Qin, Peter Calabrese, Irene Tiemann-Boege, Deepali Narendra Shinde, Song-Ro Yoon, David Gelfand, Keith Bauer, Norman Arnheim

**Affiliations:** 1 Molecular and Computational Biology Program, University of Southern California, Los Angeles, California, United States of America; 2 Program in Core Research, Roche Molecular Systems, Alameda, California, United States of America; University of Wisconsin-Madison, United States of America

## Abstract

The frequency of the most common sporadic Apert syndrome mutation (C755G) in the human fibroblast growth factor receptor 2 gene *(FGFR2)* is 100–1,000 times higher than expected from average nucleotide substitution rates based on evolutionary studies and the incidence of human genetic diseases. To determine if this increased frequency was due to the nucleotide site having the properties of a mutation hot spot, or some other explanation, we developed a new experimental approach. We examined the spatial distribution of the frequency of the C755G mutation in the germline by dividing four testes from two normal individuals each into several hundred pieces, and, using a highly sensitive PCR assay, we measured the mutation frequency of each piece. We discovered that each testis was characterized by rare foci with mutation frequencies 10^3^ to >10^4^ times higher than the rest of the testis regions. Using a model based on what is known about human germline development forced us to reject (*p* < 10^−6^) the idea that the C755G mutation arises more frequently because this nucleotide simply has a higher than average mutation rate (hot spot model). This is true regardless of whether mutation is dependent or independent of cell division. An alternate model was examined where positive selection acts on adult self-renewing Ap spermatogonial cells (SrAp) carrying this mutation such that, instead of only replacing themselves, they occasionally produce two SrAp cells. This model could not be rejected given our observed data. Unlike the disease site, similar analysis of C-to-G mutations at a control nucleotide site in one testis pair failed to find any foci with high mutation frequencies. The rejection of the hot spot model and lack of rejection of a selection model for the C755G mutation, along with other data, provides strong support for the proposal that positive selection in the testis can act to increase the frequency of premeiotic germ cells carrying a mutation deleterious to an offspring, thereby unfavorably altering the mutational load in humans. Studying the anatomical distribution of germline mutations can provide new insights into genetic disease and evolutionary change.

## Author Summary

Some human disease mutations occur 100–1,000 times more frequently than would be predicted from genome wide studies of mutation in different species. In Apert syndrome, for example, two-thirds of all new causal mutations occur at only one base in the affected gene. This unusually high frequency suggests that something about that DNA base or its local surroundings makes it highly susceptible to mutation. We studied this hypothesis by examining the location of cells containing this mutation in the testes of normal men. We found that mutant cells were not uniformly distributed throughout the testes, as would be expected for random mutations. Instead, we found 95% of the mutants in small clusters containing only a few percent of the total testes cells. A higher-than-average mutation rate could not explain the data. We propose that these mutations arise at the expected rate, but that mutated cells gain a selective advantage that allows them to increase their frequency compared to nonmutant cells. Our results—which argue against the mutational hot spot model in favor of a selection model—suggest how germline selection in animals can alter the mutational load of a species.

## Abstract 3

The frequency of Apert syndrome mutations is 100-1,000 times higher than expected from average mutation rates, and it is due to positive selection in the testis increasing the frequency of germ cells carrying the defect.

## Introduction

Current methods to measure directly the frequency at which human germline nucleotide substitutions arise each generation include the analysis of sporadic cases of human autosomal dominant or sex-linked diseases [1,2] and DNA analysis of sperm [3–5]. Studies of the fibroblast growth factor receptor 2 gene *(FGFR2)* using both approaches [3,4,6,7] have been useful in understanding the origins of Apert syndrome. Individuals born with this condition are characterized by a number of features, including prematurely fused cranial sutures and fused fingers and toes (see accession numbers). This dominantly inherited disease arises with a frequency between 10^−5^ and 10^−6^ [6,7] virtually always due to a spontaneous germline mutation,. Surprisingly, the high incidence of Apert syndrome cannot be explained by mutations at numerous nucleotide sites in the *FGFR2* gene leading to the disease phenotype. In fact, greater than 98% of Apert syndrome cases arise by transversion mutations at only two sites (C755G and C758G). The C755G mutation is located at a CpG [dinucleotide containing a C followed by a G (5′ to 3′)] site (leading to a serine-to-tryptophan substitution at amino acid 252) and accounts for two-thirds of the cases [8,9]. Based on what is known about the frequency of transversion mutations at neutral CpG sites since humans and chimpanzees last had a common ancestor [10] and mutations at many human disease loci [11], the C755G mutation frequency is 100–1,000-fold greater than expected. One intuitive explanation is that this nucleotide simply has a higher than average chance of undergoing a base substitution (mutation hot spot model). Alternatively, positive selection for diploid germline cells carrying the mutation has also been proposed [4,5,12,13]. In fact, the authors of one study [4] argued that positive selection on mutant spermatogonia was required to explain their results on sperm mutation frequencies. We show, however, that this same data is also compatible with the mutation hot spot model (Text S1 and Figure S1). With specific regard to human germline selection being responsible for the high C755G mutation frequency, it has been said that “Surprising hypotheses call for unusually strong evidence” [14]. We have therefore developed a new approach to distinguish between the selection and hot spot models that goes beyond sperm analysis.

## Results

### Analysis of Individual Testes and Their Associated Epididymides

**Figure 1:**
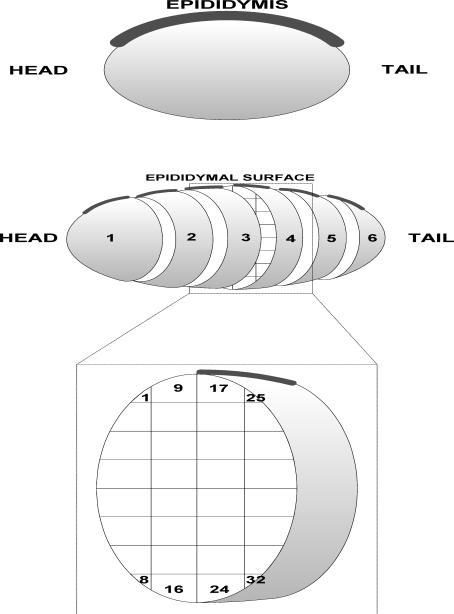
Testes Dissection Strategy Testes 374–1, 374–2, and 854–2. After slicing each testis in half, perpendicular to the epididymal axis, the two halves were each divided into three slices along the same axis for a total six slices. Each slice is then cut into 32 pieces and each piece is numbered (see inset for slice 3) to provide a binomial classification system (e.g., slice 3 piece 17). Note that there is some variation in slice and piece size because of the shape of the testis. This is reflected in the number of genomes per piece (see Table S1).

We studied the spatial distribution of C755G mutations within both testes from each of two normal individuals. The source of the testes, their dissection into small pieces, the purification and quantitation of DNA, and the quantitative mutation detection assay are described in Materials and Methods, Figure 1, Text S1, and Figures S2–S4. For testis 374–1, the mutation frequencies of 192 individual pieces ranged from <10^−6^ (no mutants found among one million genomes) to as high as 0.027 with an average frequency of 3.8 × 10^−4^ (Table 1). This latter value was close to that observed for an epididymal sperm sample taken from the proximal vas deferens and distal epididymis of the same testis (4.5 × 10^−4^). The background mutation frequency of this assay was between 2.4 × 10^−7^ and 6.6 × 10^−8^ (see Materials and Methods).

**Table 1:**
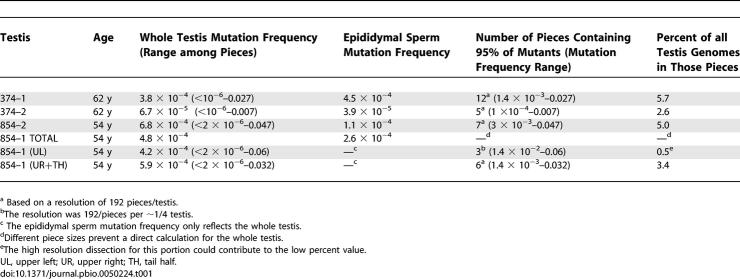
Summary Data on Four Testes

For testis 374–2, the mutation frequency for the individual pieces varied from <10^−6^ to 0.007. The average frequency (6.7 × 10^−5^) was lower than that of testis 374–1 but was consistent with the epididymal sperm sample (3.9 × 10^−5^) for this testis. The detailed data on the total DNA content and mutation frequency of each piece for these two testes (and the two additional testes examined below) are presented in Table S1. When the data from testes 374–1 and 374–2 are combined, the estimated sperm mutation frequency for this donor is similar to the highest sperm mutation frequencies of normal men of similar age using sperm as a DNA source [3,4] (unpublished data).

**Figure 2:**
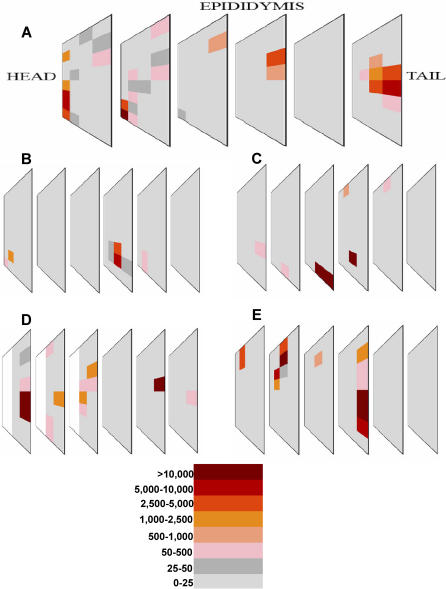
Distribution of C755G Mutants in Dissected Human Testes For dissection details, see Figure 1 and Figure S2. In every case, the orientation of the testis is given relative to the head and tail portions of the epididymis. Each panel consists of six slices, each divided into 32 pieces, with exceptions noted below. The color code shows the number of mutant molecules per million genomes. (A) Testis 374–1. (B) Testis 374–2. (C) Testis 854–1, Upper left (UL) portion (Figure S2). The sizes of the 192 pieces are ∼1/4 the size of the pieces shown in the other panels. (D) Testis 854–1 Upper right + Tail half portions (UR+TH). The vertical white rectangles (pieces 1–16 in the first three slices) indicate the source of the UL portion shown in panel (C). (E) Testis 854–2.

Figure 2A and 2B shows the distribution of the mutation frequencies throughout both testes. Each one is characterized by a very small number of “hot” pieces with very high mutation frequencies compared with the mutation frequencies of the remaining pieces. We also calculated the minimum number of pieces which together contained 95% of the mutant genomes. In testis 374–1, 12 of the 192 pieces satisfied this criterion. These 12 pieces contain only 5.7% of the DNA in this testis, whereas we might have expected that according to a random spatial distribution, many more pieces which together contain 95% of the genomes would have been required. The results for testis 374–2 were quite similar: of the 192 pieces, 95% of the mutant molecules came from five pieces whose total DNA content was only 2.6% of the total testis. In many cases, several pieces with high mutation frequencies appear to form foci adjacent to one another in one slice or even between slices (Figure 2A and 2B).

### Analysis of a Second Pair of Testes Partially at Higher Resolution

We first analyzed the mutation frequency distribution in a portion (approximately one-quarter) of testis 854–1. By dissecting this portion into 192 pieces (UL in Figure S2), we achieved a resolution four times higher than used for testes 374–1 and −2. The mutation frequencies of the pieces varied from <2 × 10^−6^ to 0.06. We found that 98% of the mutants were derived from only three of the 192 pieces (Figure 2C). These pieces contained 1.6 % of the genomes in this portion of the testis. The fact that the pieces with the highest mutation frequencies were approximately one-quarter the size of pieces analyzed before suggests that the “hot” pieces from testes 374–1 and −2 studied at the lower resolution might have contained sub-regions with even higher mutation frequencies.

The remaining three-quarters of testis 854–1 was analyzed with the same dissection resolution (Figures 1 and S2) used for donor 374 and therefore was cut into 144 pieces (=0.75 × 192). The mutation frequencies for individual pieces ranged from <2 × 10^−6^ to 0.032. Again, the vast majority of the mutations (95%) came from a few (six) pieces that contained only a small fraction of the genomes in this portion (5%). The mutation distribution is shown in Figure 2D. The total mutation frequency of testis 854–1 calculated using all the data (4.8 × 10^−4^) was quite similar to the epididymal estimate (2.6 × 10^−4^).

Finally, we examined testis 854–2 in the same way as 374–1 and 374–2. This testis had the same features found in the other three: a similar distribution (Figure 2E) and range (Table 1) of mutation frequencies and an enrichment of mutant genomes in the hottest pieces (95% of the mutants come from 2.6% of the testis genomes). Surprisingly, the total mutation frequency of this testis was quite different from the epididymal estimate (Table 1). This epididymal sperm DNA preparation was unique in containing a highly viscous material, suggesting that the epididymis might have been subject to some local pathological process that contaminated the sperm sample with nongermline DNA, thereby explaining the lower mutation frequency estimate.

In summary, analysis of the distribution of C755G Apert syndrome mutations in four testes showed a small number of pieces with very high mutation frequencies among a general background of lower mutation frequencies. Some of the pieces had 10^3^- to >10^4^-fold higher mutation frequencies than pieces with the lowest frequencies. In many cases, several pieces surrounding “hot” regions also had relatively high mutation frequencies.

### Testing the Mutation Hot Spot Model

**Figure 3:**
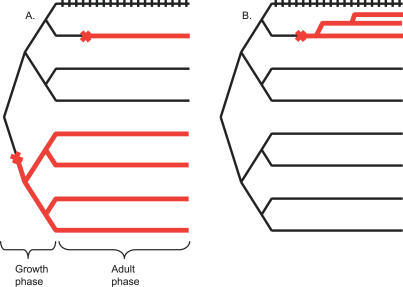
Model Schemes The models are depicted with only three growth phase generations leading to eight adult SrAp cells; in actuality there are ∼30 growth phase generations leading to ∼10^10^ adult SrAp cells. The vertical tick marks across the top line depict adult phase generations; for the donors in this paper there are ∼1,000 adult phase generations. (A) Mutation hot spot model (*p* = 0). There are two mutation events (red crosses): one is in the adult phase which produces one mutated SrAp cell, while the other one in the growth phase produces four mutated SrAp cells. (B) Selection model (*p* > 0). The one mutation in the adult phase now produces three mutated SrAp cells.

To understand whether the unexpectedly high C755G transversion mutation frequency can be explained simply by an inherently higher probability of mutation at this site, we asked whether the frequencies and distribution of the C755G mutations we observed in the testes can be explained by a plausible model of germline mutation (described in the Materials and Methods section). In this model, we make use of data on human germline development and maturation[1,15–28]. Our model has two phases (Figure 3). In the first phase between zygote formation and puberty, called the growth phase, the male germline cells divide symmetrically and thus increase in number exponentially. At each division, there is a probability of a mutation at the disease site. The exponential increase in cell number can magnify the influence of any single mutation on the mutation frequency if it occurs early enough in development. This concept, first noted by Luria and Delbruck [29] in bacteria, can lead to a “mutation jackpot.” Because the germ cells would be expected to stay in close proximity to their ancestors, any such early mutations will result in regions of the testis with high mutation frequencies. The germ cells of the growth phase eventually form the adult self-renewing Ap spermatogonia (SrAp). In the second phase, called the adult phase, SrAp divide asymmetrically to produce a daughter SrAp (self-renewal) and another daughter cell, whose descendants, after only a few additional divisions, will produce sperm. During the adult phase, the number of SrAp remains approximately constant, and each new mutation in this phase produces only one mutated SrAp cell lineage. There are many more SrAp cell divisions during the adult phase than there are SrAp precursor cell divisions during the growth phase; for a 50–60-y-old man, the ratio of mutations expected to occur in the adult phase, compared to the growth phase, is approximately 500 to 1 (see Materials and Methods).

We have written a computer program to simulate the model (Protocol S1). The code can also be downloaded from http://rd.plos.org/pbio.0050224. This program allows us to estimate model parameters and test the hypothesis that the data are consistent with this model. A key model parameter is the nucleotide substitution rate per cell division. We infer this value by finding that rate that, in simulations, is most likely to match the overall C755G testis mutation frequency. Even though this frequency is very high relative to data on transversion frequencies at other CpG sites [10,11], the inferred C755G mutation rate per cell division is still low enough that mutations early in the growth phase with the potential to produce a jackpot are unlikely. In those simulations with this inferred rate, almost all of the mutations occur in the adult phase or late in the growth phase, and consequently, there is little variation in the mutation frequency of the testis pieces: 95% of the mutants are found distributed among 95% of the testis pieces. This contrasts sharply with the experimental data where the mutation frequency varies greatly between testis pieces, and 95% of the mutant genomes are found in only a few testis pieces containing between 2.6% and 5.7% of the total genomes. In simulations, when we increase the mutation rate per cell division so that more mutations occur in the growth phase, there are so many mutations in the adult phase that the overall testis mutation frequency is much greater than that found in the actual data.

To test whether the observed distribution of the mutation frequencies among the testis pieces is consistent with the model, we performed both a goodness-of-fit test and a chi-squared test (see Materials and Methods). The mutation hot spot model was rejected (*p* < 10^−6^) (Table 2). We reached the same conclusion when we considered a model with replication-independent mutations (or a model with both replication-dependent and replication-independent mutations), since the adult phase lasts much longer than the growth phase (see Materials and Methods). Our data provide strong support for rejecting the most intuitive explanation for the high frequency of C755G mutations: that the site has an inherently higher probability of undergoing transversion mutations than does an average CpG site in the genome. We conclude that this site is not a mutation hot spot, regardless of the exact molecular mechanism responsible for the C755G mutation event.

**Table 2:**
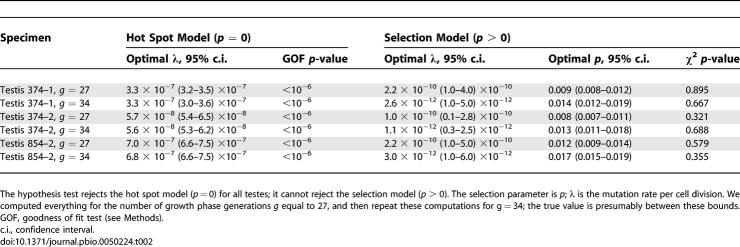
Model Parameters and Goodness-of-Fit *p*-Values

### Germline Selection

Incorporating selection as a modification to our model can reproduce both the overall testis mutation frequency and the distribution of mutation frequencies observed in the testis pieces. One possibility is that the C755G mutation promotes rare SrAp symmetric divisions in the adult phase (in the original model, adult phase divisions were always asymmetric [16–18,21,24]). The occasional symmetric divisions would allow mutated SrAp to grow locally in number over time, and thereby increase the overall mutation frequency in the testis. We add to our first model a selection parameter: at each adult phase generation, with probability *p*, a mutated SrAp divides symmetrically and with probability 1− *p* it divides asymmetrically (after a symmetric division, each daughter SrAp reverts to having asymmetric divisions until the next rare symmetric division occurs; a similar model was independently proposed in a recent publication [12]).

We infer the probability *p* and the mutation rate per cell division by fitting both the overall testis mutation frequency and the minimum number of pieces, which together contain 95% of the mutant genomes. The inferred probability *p* of a symmetric division is approximately 0.01 (on average, one of every hundred divisions is symmetric). With this modification, we can no longer reject the model using the chi-squared test (Table 2): the distribution of frequencies in the testis pieces now matches the data. In the simulations, foci of high mutation frequency emerge and, as in the data, these foci often intersect several adjacent testis pieces. With *p* near 0.01, simulations with the inferred mutation rate match the observed mutation frequencies of the testes (10^−4^–10^−5^); however, simulations with this rate, but with *p* = 0 (the no-selection case), predict a testis mutation frequency in the 10^−8^–10^−10^ range for a 20-y-old male. This lower range is similar to C-to-G mutation frequencies estimated for CpG sites in the studies on neutral and disease mutations [10,11].

Another difference between the *p* = 0 (no-selection) and the *p* > 0 cases is that for the *p* = 0 case, the model predicts that the mutation frequency increases linearly with the man's age, whereas for the *p* > 0 case, this increase is exponential. Previously, it was suggested [4] that selection could cause the observed exponential increase in the birth incidence of Apert syndrome with the father's age (paternal age effect [1,12,15,30,31]). To match the observed 20-fold increase between fathers who are younger than 24 and fathers who are older than 50 [30], the inferred *p* value would have to be approximately 0.004.

There are undoubtedly other models with different forms of selection on the mutant cells that are also consistent with the data. A modification to the model that does not include selection, but could reproduce the testis data, is if there was a higher nucleotide substitution rate per cell division in the growth phase than in the adult phase. However, this could not account for the paternal age effect, nor do we know of any evidence to support such a difference.

### Testis Distribution of a C-to-G Transversion Mutation at a Control CpG Site

As a control, we also analyzed the C of a CpG site in an intron of the *CAV1* gene on Chromosome 7, which is presumably a neutral site. We reanalyzed testis 374–1 and 374–2 using an assay designed for this site (Materials and Methods). The data are given in Table S2. The total C-to-G mutation frequency in sperm from epididymis 374–1 was 7 × 10^−6^. The mutation frequency that was estimated by summing up the individual pieces of the testis was 3.3 × 10^−6^. Unlike the disease site, there was a narrow range of frequencies in these individual pieces (<4 × 10^−6^ to 2 × 10^−5^). Ninety-two testis pieces were required to collect 95% of the mutants; these pieces contained 50% of the testis' genomes. Eighty-eight pieces, containing 46% of the genomes, had mutation frequencies less than the background of the assay. Therefore for the testis pieces contributing to the mutation estimate, 88% of the pieces, containing 93% of the genomes, were required to amass 95% of the total mutations. Moreover, selection is not required to explain the data, since we could not reject our model for the neutral mutation case with the selection parameter *p* fixed at zero. The data from testis 374–2 was similar.

The control site mutation frequency is two orders of magnitude less than at the disease site; it is somewhat higher than expected from other estimates [10,11], but those estimates are presumably from younger reproducing-age individuals, whereas donor 374 is 62 y old and has had approximately seven times more adult phase generations to accumulate mutations. The lack of any foci with very high mutation frequencies suggests that Luria and Delbruck [29] jackpot formation is rare in the human testis, and that something is fundamentally different between this CpG site and the Apert 755 disease CpG site.

## Discussion

In theory, heritable nucleotide substitutions can arise in any germline cell during scheduled DNA replication or in nondividing cells by means of error-prone DNA repair (see [32]). Two observations support the importance of the cell division–dependent mutation process. First, neutral germline mutations seem to arise 3–6-fold more frequently in males than females in many organisms, including humans (male-driven evolution, see [33]). Second, a number of genetic conditions including Apert syndrome appear to increase in the offspring of men as they age (paternal age effect [1,12,15,30,31]). Both male-driven evolution and the paternal age effect can be explained by the cell division–dependent mutation process, because cell divisions of self-renewing spermatogonia occur throughout a man's life, whereas the cellular precursors of eggs (oogonia) cease replication during the fetal life of a female [1,15].

We examined the molecular anatomy and frequency of the Apert syndrome C755G mutation in normal human testes to test whether the high mutation frequency was due to an exceptionally high C-to-G transversion mutation rate per cell division. The results show that the observed C755G mutation frequency and distribution within the testes cannot be explained by this hot spot model (*p* < 10^−6^).

An alternate hypothesis to explain the high C755G mutation frequency argues that diploid premeiotic cells carrying the C755G mutation have a selective advantage over wild-type cells [4,5,12,13]. In one case [5], the authors cited the puzzling observation that the magnitude of the sex bias for the C755G Apert syndrome is at least 99-fold greater in the male germline than in the female germline [34,35], whereas estimates of male bias (male-driven evolution) using data on neutral mutations at many different sites would indicate only an ∼5-fold male preference [33,36,37]. Rare patients with multiple mutations in the *FGFR2* gene that leads to Apert syndrome were also cited as support for a germline selection model [13]. Finally, in another study [4], the authors exploited a nearby single nucleotide polymorphism (SNP) to argue that selection acted on the C755G mutation (however, see Text S1 and Figure S1).

In our experiments, we were not only able to clearly reject the hot spot model, but also we could show that modifying our model of germline development by incorporating a simple selection scheme led to predictions on mutation frequency and testis distribution consistent with our data. This selection takes place on SrAp cells carrying the C755G mutations that arise at approximately the frequency expected from the existing data on neutral mutations [10,11]. The selection model proposes that mutant adult SrAp occasionally divide symmetrically (inferred rate 1 out of 100 divisions on average, or approximately once every 4 y), whereas wild-type SrAp always undergo asymmetric self-renewal divisions. Considering all of the published work [4,5,12,13] as well as our present results, it now seems very likely that positive selection can be a driving force acting to increase the germline mutation frequency in humans above the frequency at which spontaneous nucleotide substitutions arise.

We would like to emphasize that the type of selection we are discussing is on diploid premeiotic cells. Previous proposals have suggested that C755G mutation bearing sperm may have a selective advantage over wild-type sperm [3,^38^]. Selection taking the form of competition among sperm is well known in plants and animals [39], and is even documented in primates [40]. However, for this particular mutation, the testis and epididymal sperm data we collected as well as data on ejaculated sperm ([3,4] and unpublished data) and Apert syndrome birth data [6,7] show similar mutation frequencies. Therefore, while further selection on sperm is possible, we believe that the vast majority of the increase in mutation frequency is due to selection on the diploid premeiotic cells.

Why should a mutation that has a distinct selective disadvantage when present in all the cells of an organism have a selective advantage when present only in a small fraction of the germline cells? It is worth noting here that achondroplasia, the most common cause of dwarfism, has many similarities to that of Apert syndrome (see [5]). Virtually all of the new achondroplasia mutations arise in the male germline at one nucleotide site (G1138A) in the fibroblast growth factor receptor 3 *(FGFR3)* gene and with a mutation frequency even higher than the C755G mutation in *FGFR2*. These common characteristics suggest that the G1138A mutation may also increase to such a high frequency by a selective mechanism [4,5,12,13]. It is interesting to note that both FGFR2 and FGFR3 are receptor tyrosine kinases and can influence downstream members of the signal transduction pathway (for a more detailed discussion see [41,42]). Especially relevant may be the fact that some mutations in *FGFR2* and *FGFR3* (although usually not the specific mutations that cause Apert syndrome or achondroplasia) have been associated with certain cancers [43,44] and cancer susceptibility [45].

Germline selection in diploid germ cells of animals was considered by the population geneticist Ian Hastings [46,47]. He examined how mitotic gene conversion and somatic crossing over events in diploid germline cells of animals could lead to loss of heterozygosity of recessive alleles and the possibility of positive or negative selection on such alleles. He calculated that selection against rare germline cells made homozygous for a recessive allele can effectively lower the transmission of the deleterious allele to offspring thereby reducing disadvantageous alleles entering the population and burdening it with reduced viability or fertility. Using plausible models, this reduction in the mutational load could be as large as 100-fold. Similarly, loss of heterozygosity could allow recessive alleles that conferred a germline advantage to be spread more quickly in the population. Experimental literature on germline selection in premeiotic diploid cells in animals is very sparse (see [48] and references therein) but in one case, wild-type *Drosophila* cells were produced by a genetic trick in the germline of females heterozygous for a phenotypically recessive mutation and were found to have a proliferative selective advantage compared to the background heterozygous cells.

Hastings' analysis was primarily concerned with recessive alleles that were already polymorphic in the population. But gain-of-function mutations that arise sporadically in the testis would behave in the same way, because a second event leading to loss of heterozygosity is not required for positive and negative selection to be effective. A new gain-of-function mutation with a germline selective advantage will more likely be transmitted to the next generation, because the effective mutation frequency is elevated beyond the level that can be achieved by the mutation process alone. A disadvantageous gain of function mutation would be less likely to be tested in the population if it were selected against in the germline. Finally, Hastings realized that alleles conferring a selective advantage in the germline may be disadvantageous in the adult and might lead to “mitotic drive” systems that could increase the mutational load of a population. Both Apert syndrome and achondroplasia may be examples of such a system, and additional examples of mutations of medical interest may also exist (see [15,30,31,33]). The method we have developed can be used to test this hypothesis at any locus in many different species if a sufficiently sensitive mutation assay can be made available.

## Materials and Methods

### Source of testes.

Both testes from each of two donors (374, 62 y old, and 854, 54 y old) were supplied by the National Disease Research Interchange (NDRI) in Philadelphia, PA, United States. The testes were frozen no longer than 10 h post mortem and stored at −80 °C. Procurement of the testes from the NDRI was approved by the Institutional Review Board of the University of Southern California.

### Testis and epididymis processing.

The epididymis was removed from the testis. Sperm cells were collected from the tail of each epididymis and the first ∼3 cm of the adjacent vas deferens after they were cut into small pieces and incubated in 25 ml of 2.2% sodium citrate with shaking for 60 min at room temperature. Sperm cells were concentrated by centrifugation at 6000*g*. Sperm DNA was isolated using a Puregene DNA purification kit and protocol (Gentra Systems; http://www1.qiagen.com/Products/dna.aspx) except that 40 mM DTT was added to the lysis solution. Each testis was fixed in 70% ethanol at 4 °C for about 3 d. Three of the four testis (374–1, 374–2 ,and 854–2) were each cut into six slices and each slice further divided into 32 pieces. The dissection scheme and how the position of each piece within the testis was recorded is shown in Figure 1. Figure S2 shows the slightly different scheme that was used only on testis 854–1. An introduction to testis anatomy can be found at: http://training.seer.cancer.gov/ss_module11_testis/unit02_sec01_anatomy.html. DNA was extracted using a Puregene kit (Gentra Systems). DNA concentrations were estimated using real-time PCR and primers for a unique sequence on Chromosome 21 (additional details are found in Text S1). The number of genomes estimated for each testis piece is presented in Table S1.

### Measurement of the *FGFR2* C755G mutation frequencies in testis and epididymal sperm DNA samples.

The assay for C755G mutations resembles an allele-specific PCR (ASPCR) that amplifies mutant but not wild-type molecules using a primer whose 3′ end contains a base that is complementary to the mutant [49–51]. The most critical difference between ASPCR and the method we used (pyrophosphorolysis-activated PCR, or PAP [52,53]) is that both PAP primers are blocked at their 3′ ends with a dideoxynucleoside monophosphate (ddNMP). The PAP primers anneal perfectly to the mutant template but anneal to the wild-type template with 3′ terminal mismatches (Figure S3). Primer extension by DNA polymerase, and thus PCR, is not expected to occur in either case. However, we used a DNA polymerase (in our case TMA31FS [54]) capable of both efficient pyrophosphorolysis of ddNMP terminated primers (thereby unblocking them) and primer extension using dNTPs. The pyrophosphorolysis and amplification preferentially occurs using the mutant templates. PAP is far more selective than conventional ASPCR for rare mutation detection. If the TMA31FS polymerase mistakenly removes a mismatched terminal ddNMP, insertion of the correct (templated) nucleotide simply results in the generation of another copy of the wild-type template and not loss of selectivity. To measure the mutation frequency of a DNA sample, 25-μl PAP reactions were carried out in 96-well plates [Opticon 2, MJR (BioRad; http://www.biorad.com)] or 10-μl reactions in 384-well plates [ABI 7900HT (Applied Biosystems; http://www.appliedbiosystems.com) or Roche LightCycler 480 (Roche Applied Science; http://www.roche.com)]. Each reaction contained 20 mM HEPES (pH 7.5), 30 mM KCl, 50 μM Na_4_PPi, 2 mM MgCl_2_, 80 μM of each dNTP, 80–200 nM of each primer, 2 μM Rox, 0.2 X Syber Green I, 0.04 unit/μl TMA31FS DNA polymerase (Roche Molecular Systems), and 25,000 copies of testis or epididymal sperm DNA molecules. Research samples of TMA 31FS DNA polymerase may be obtained from Thomas W. Myers, Director, Program in Core Research, Roche Molecular Systems, 1145 Atlantic Avenue, Alameda, CA 95401, USA (thomas.myers@roche.com). The sequences (see accession numbers) of the C755G-specific PAP primers used in these experiments are: 5'-CCCCACTCCTCCTTTCTTCCCTCTCTCCACCAGAGCGAT(_dd_G) and 5'-TTTGCCGGCAGTCCGGCTTGGAGGATGGGCCGGTGAGGC(_dd_C)._._ The 79-bp PAP product makes detection possible using quantitative PCR . The primers were synthesized by Biosource International (http://www.biosource.com) or Biosearch Technologies (http://www.biosearchtech.com). The PAP primer ending in a _dd_C was synthesized by conventional methods. The PAP primer ending with _dd_G required synthesis using 5'-CE phosphoramidites. The _dd_NTP at the 3' end of each primer is complementary to nucleotide 755 but each to different strands.

The PAP cycling conditions included an initial denaturation step of 2 min at 94°C followed by 150 cycles of 6 s at 94 °C and 40 s at 78 °C. Positive reactions could easily be distinguished from negative ones by considering the kinetics of the increase in fluorescence with cycle number and the melting profile of the final PCR product. For sample PAP data, see Figure S4.

### Measurement of the control mutation frequencies in testis and epididymal sperm DNA samples.

For the control, we measured the frequency of a C-to-G transversion at a CpG site on human Chromosome 7. The C nucleotide is known to be polymorphic in the human population (see accession numbers). Testis donor 374 was genotyped and found to be a C/C homozygote and thus his testis and sperm cells could be examined for the presence of C-to-G transversion mutations at this site. To measure the frequency of mutation, 10 μl PAP reactions were carried out in 384-well plates (ABI 7900HT, Applied Biosystems). Each reaction contained 20 mM HEPES (pH 7.5), 30 mM KCl, 50 μM Na_4_PPi, 2 mM MgCl_2_, 80 μM of each dNTP, 200 nM of each primer, 2 μM Rox, 0.2 X Syber Green I, 0.04 unit/μl TMA31FS DNA polymerase (Roche Molecular Systems), and 25,000 copies of testis or epididymal sperm DNA molecules/reaction. The sequences of the C755G-specific PAP primers are: 5′-TATTAATATAACTTAGTATCTGTCACCCCAAGGGAACCAA(_dd_G) and 5′-TATTAATATAGAGTATTGACTCTTATTCTTGGGCTTCGAC(_dd_C). Note that the ten 5'-most nucleotides of these primers are not complementary to the mutated target sequence. An 81-bp PAP product results. The PAP cycling conditions included an initial denaturation step of 1 min at 94 °C followed by 134 cycles of 6 s at 94 °C and 40 s at 70 °C.

### Mutation counting strategy.

A total of 40 reactions containing 25,000 genomes each (a total of 10^6^ genomes) were used to estimate the C755G mutation frequency for every piece of testes 374–1 and −2. In those cases where fewer than 25/40 reactions were positive, we took the number (after Poisson correction) as an estimate of the C755G mutation frequency for that piece. If 25 or more reactions were positive, then the experiment was repeated using 10-fold dilutions until a dilution of the DNA from the piece was found to give fewer than 25/40 positives.

Based on the mutation frequency results from the first pair of testes, a slightly different strategy was used for testes 854–2 and the upper right and tail half (UR and TH, respectively) parts of 854–1 (Figure S2). First, ten reactions were carried out on every piece (total of 250,000 genomes analyzed). In the case of those pieces where four or fewer reactions were positive, we took the number (after Poisson correction) as an estimate of the C755G mutation frequency. In those cases where five or more of the ten reactions were positive, we carried out additional experiments using 40 reactions (1,000,000 testis genomes) and appropriate dilutions in the manner described for the 374 testes. Repeated measurements from the same piece had an average deviation of 32% from the mean value.

For all mutation counting, the estimate of the total testis mutation frequency was the average of the frequencies of the pieces weighted by the number of genomes in those pieces. For a positive assay control, ten or 20 Apert C755G mutant molecules from a patient heterozygous for the C755G mutation (kindly provided by Dr. Mimi Jabs, Johns Hopkins Medical Institutions) were added to 500,000 human genomes and divided into 20 reactions (an average of 0.5 or 1 mutants per reaction). Negative assay controls consisted of 25,000 genomes of nongermline human blood DNA from normal individuals (Clontech; http://www.clontech.com). Over the course of our study, we observed an assay background in human blood DNA of between 6.6 × 10^−8^ and 2.4 × 10^−7^, based on experiments using ∼67,000,000 control genomes. This places an upper limit on the possible mutation frequency in human white blood cells, although it would be lower if false positives can also result from artifacts of the assay itself.

The method for counting the Chromosome 7 control C-to-G transversion mutations was identical to that used for testes 854–2 and the UR and TH parts of 854–1. For a positive control, 20 mutant genomes of blood DNA from a heterozygous (C/G) individual were added to 500,000 human genomes from individuals homozygous for the C allele and divided into 20 reactions (an average of one G allele-containing genome per reaction). Negative controls consisted of 20 reactions (a total of 500,000 genomes) of blood DNA from individuals homozygous for the C allele.

### Biological basis for the model.

Classical cytological approaches provide our main source of information about human germline development and maturation [1,15–28]. Not long after fertilization, human primordial germ cells arise in the developing human embryo and migrate to the embryonic sites of testis formation (genital ridges). The germ cells become organized into seminiferous tubules, proliferate, and differentiate into gonocytes and fetal spermatogonia. By the beginning of puberty, the seminiferous tubules are composed primarily of spermatogonia and supporting Sertoli cells. The spermatogonia (including the adult SrAp) are located in their niche at the periphery of the tubule and in intimate association with Sertoli cells. After puberty, seminiferous tubules also contain B spermatogonia [17] that are derived from the asymmetric division of SrAp. B spermatogonia are a precursor of meiotic cells. Four B spermatogonia and eventually 16 sperm are thought to result from each division of one SrAp [16,17,21,24]. In spermatogenesis, both meiotic and post-meiotic cells gradually move from the periphery toward the lumen of the tubule. Sperm enter the lumen of the tubule (spermiation) and are transported to the epididymis, where they undergo further development and are stored. Note that in a seminiferous tubule segment, any mutant SrAp will be in close proximity to its spermatogonial, meiotic, and post-meiotic cell descendants that will carry the same mutation. Although SrAp divide every 16 d, it takes much longer than 16 d for the division products of one to yield sperm [18]. This creates overlapping “generations” of different germline cell types that are found in close proximity to one another and all derived from the same SrAp cell.

### Quantitative modeling.

We model the testis in two phases (Figure 3). In the first phase between zygote formation and puberty, called the growth phase, self-renewal cells divide symmetrically to produce two self-renewal cells. This continues for *g* generations. In this phase, the testis grows exponentially from one self-renewal cell to 2*^g^* self-renewal cells. The total number of divisions in the testis in this phase is (2*^g^* − 1). This corresponds to a total of (2*^g^* ^+1^ − 2) daughter cells being produced, and there are this many opportunities for replication-dependent mutations. There is a mutation rate per cell division λ at the disease site. A mutation is shared by all of a cell's descendants, so a mutation early in the growth phase will produce many mutants. To be more specific, a mutation at the *k*th generation will produce 2*^g^* ^−*k*^ mutant descendants. This concept, first noted by Luria and Delbruck [29] in bacteria, can lead to a “mutation jackpot.” Since the germ cells would be expected to stay in close proximity to their ancestors, any such early mutations will result in regions of the testis with high mutation frequencies. We will discuss the spatial details in the Spatial Structure section below.

Zhengwei et al. measured on average 656,000,000 type A spermatogonial cells per testis [23]. Using the number of genomes per testis we have measured, the distribution of the different cell types in Table 2 of [23] (to add sperm, see [20]), and the ploidy of these different cell types, we infer that for donor 374, each testis has approximately 480,000,000 type A spermatogonial cells and that for donor 854, each testis has approximately 260,000,000 type A spermatogonial cells. Because one-half of the type A spermatogonial cells are SrAp [20,24,55], we estimate the number of growth phase generations *g* is 27 or 28. We have also considered higher numbers from the literature (30 [15] or 34 [19]).

In the second phase, called the adult phase, SrAp divide asymmetrically to produce one SrAp and another cell whose descendants will ultimately produce sperm. In this phase, the number of SrAp remains constant. These cells divide every 16 d [18], so assuming this phase begins at puberty (age 13), donor 374 who is age 62 has experienced *a* = 1,127 adult phase generations, and donor 854 who is age 54 has experienced *a* = 943 adult phase generations. The total number of divisions in the testis in this phase is equal to (*a* × 2*^g^*). For these donors, there are approximately 500 (≈a × 2*^g^*/2*^g^* ^+1^) more opportunities for replication-dependent mutation in the adult phase than in the growth phase, and because we assume identical mutation rates per cell division in both the growth and adult phases, there will also be, on average, this many more mutations in the adult phase. These mutations will be randomly spread throughout the testis. Unlike in the growth phase, mutations in the adult phase lead to only a single mutated SrAp (and approximately 25 other mutated germ cells [23]). If the testis mutation frequency is *f*, then *f* ÷ *a* is an upper bound for the mutation rate λ (this inequality ignores mutations in the growth phase).

We examine two modifications to this model. First we consider replication-independent mutations. The number of these mutations is proportional to time rather than the number of divisions. The literature is not definitive [27,28], but it appears that most of the growth phase generations occur in approximately 1 y (the 9 mo of embryonic and fetal development plus possibly several months of infancy). There may be several further growth phase generations in the ∼13 y before the adult phase begins; however, the number of such generations is small enough that any mutations in these years, like in the adult phase and unlike in the growth phase, will not produce significant mutation clusters (in the absence of selection). Then, letting *z* be the age of the donor, the ratio of replication-independent mutations that cannot cause significant mutation clusters to those mutations that will cause such clusters is (2*^g^* × *z*)/(2*^g^* ^+1^/*g*). For our donors, this ratio is at least 700 to 1, even more extreme than for the replication-dependent mutations. Consequently it would be difficult for a model incorporating replication-independent mutations to explain our testis data.

The second modification we consider is selection. We introduce a new parameter into the model: at each adult phase generation, with probability *p*, a mutated SrAp divides asymmetrically, and with probability 1 − *p* it divides symmetrically to produce two mutated SrAp. This symmetric division is like that in the growth phase, except that afterward, each daughter SrAp reverts to having asymmetric divisions until the next rare symmetric division occurs; a similar model was independently proposed in a recent publication [12]. The occasional symmetric divisions allow mutated SrAp to grow locally in number over time, and thereby increase the overall mutation frequency in the testis. On average, a mutation in this phase will leave

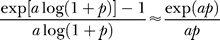
descendants (if a mutation occurs with *x* generations remaining, it leaves on average (1 + *p*)*^x^* descendants, the time of a mutation is uniform on [0, *a*]). Since the mutation probability and *p* are small, the number of self-renewal cells will still stay roughly constant in the adult phase.

### Spatial structure.

Above we discussed the number of mutant cells with regard to our various models, now we will discuss their spatial distribution. For simplicity, we have assumed that the testis is in the shape of a rectangular box, and it is divided into a regular grid of 6 × 8 × 4 = 192 cubic testis pieces (Figure 1). If a mutation event occurs in the growth phase after testis formation, then there is a mutation cluster where all of the SrAp cells in a cubic region are mutants. Depending on the size of this cluster, it may be contained within one of the testis pieces, intersect parts of several adjacent testis pieces, or contain all of one or more testis pieces and intersect parts of the surrounding testis pieces. The number of mutants in a cluster depends on the cell generation in which the mutation event occurred and is discussed in the previous paragraphs. The size of a cluster is calculated assuming that it has the same density of SrAp cells as the rest of the testis, and the total testis has 2*^g^* SrAp cells. We set the units so that 1 is the length of one of the 192 testis pieces. Then a mutation at the *k*th generation will produce a cubic mutation cluster with length (192 × 2^−*k*^)^1/3^. To consider some examples, a mutation at the first generation means half of the testis is mutated, a mutation at the 8th generation produces a cluster with 91% of the length and 75% of the volume of one of the testis pieces, a mutation at the 21st generation produces a cluster with 5% of the length and 0.01% of the volume of one of the testis pieces. The location of a cluster is random within the testis.

We assume that at the beginning of the adult phase, there is an equal fraction of the 2*^g^* SrAp cells (mutated or not) in each of the 192 testis pieces. During the adult phase, mutation events occur randomly throughout the testis. If *p* = 0, then each such mutation event produces only one mutant SrAp cell. If *p* > 0, then each mutation event produces a cluster of mutant SrAp cells. The number of mutants in a cluster is discussed in the previous paragraphs. For simplicity, we assume that the shape of a cluster is a cube. The size of a cluster is calculated assuming that the cluster has the same density of SrAp cells as the rest of the testis. Depending on the cluster's size, it may be contained within one testis piece or intersect several adjacent testis pieces. Unlike the growth-phase clusters, the SrAp cells in the selection clusters are in addition to the nonmutated SrAp cells in the testis pieces. Due to the exponential growth of these selection clusters, there is a relatively narrow range of realistic parameter values for *p*. For example, a mutation early in the adult phase will produce a cluster approximately 10^−5^ the size of the testis if *p* is 0.01, whereas this same cluster will be larger than the rest of the testis if *p* is 0.03 or greater. Multiple mutation clusters may intersect the same testis piece and a given cell may only be mutated once (there are no reverse mutations).

### Testing the model.

We have written a computer program (Protocol S1 or download from http://rd.plos.org/pbio.0050224) to simulate the model and its modifications. We fix the parameters *a* = 1,127 for donor 374 and *a* = 943 for donor 854, and consider both the extremes *g* = 27 and *g* = 34. First we consider the mutation hot spot model (*p* = 0). We simulated the model many times to estimate the probability that the simulated mutation frequency is within 5% of the observed mutation frequency as a function of the mutation parameter λ (mutation rate per cell division). The λ that optimizes this quantity is our approximate maximum likelihood estimate (this technique is sometimes called a rejection sampler) and standard methods can produce approximate 95% confidence sets. For the selection model (*p* > 0), there are two parameters to consider: *p* and λ. There are an infinite number of pairings of these parameters' values (a subset of parameter space) for which the model can match the observed mutation frequency. Consequently, we searched for the parameter values which optimized the following two-criteria probability: (a) that the simulated mutation frequency is within √5% of the observed frequency, and (b) that the simulated fraction of testis' genomes in the fewest number of testis' pieces necessary to contain 95% of the mutants is within √5% of this observed quantity. Note that for the mutation hot spot model (*p* = 0) this two-criteria probability is 0 for all values of λ, since no single value of λ can match both criteria. In general, to estimate the empirical likelihood, we simulated the model 1,000 times for each set of parameter values.

To determine whether the distribution of mutations in the 192 pieces is consistent between the model and the data, we used two hypothesis tests. First we counted the number of pieces with mutation frequencies in each of the eight color categories in Figure 2 ([0–25/million], etc.). For both the mutation hot spot model (*p* = 0) and the selection model (*p* > 0), we simulated the model under optimal parameters and counted the number of pieces with frequencies in these same categories, and then performed the chi-squared test. For the control site, since the observed frequencies are much lower, we considered the following four categories: [0–4 × 10^−6^), [4 × 10^−6^–9 × 10^−6^), [9 × 10^−6^–1.4 × 10^−5^), [1.4 × 10^−5^+). The notation [*x*–*y*) means frequencies *f* such that *x* ≤ *f* < *y*.

Because a mutation cluster can overlap adjacent testis pieces, this dependence makes the chi-squared test anticonservative. In order to not reject the selection model (*p* > 0), this test is sufficient; however, in order to reject the hot spot model (*p* = 0), we have also performed a goodness-of-fit test. For each simulation, we computed the following statistic: the minimum fraction of testis pieces which contain at least 95% of the total mutants. We simulated the hot spot model under the optimal parameters and recorded this statistic for those simulations such that the total testis mutation frequency was within 5% of the real data. We repeated this procedure such that 1,000,000 simulations met this criterion (under optimal parameters over 90% of simulations met the criterion). The simulated statistic was never lower than 90%, in contrast with the much lower values for the real data in Table 1 (<6%). Therefore an empirical *p*-value is <10^−6^. However, this value is just a function of our patience in running simulations; we contend that for however many simulations are run, this statistic will never be lower than for the data. In order to get a *p*-value independent of the number of simulations, we fit the distribution of simulated statistics to a *t*-distribution (admittedly a poor fit) and the statistics for the real data were over 300 standard deviations away, making the *p*-value less than 10^−200^. The (anticonservative) chi-squared test rejected the hot spot model with *p*-value less than 2 × 10^−16^. Regardless of which *p*-value is used, there is strong support to reject the hot spot (*p* = 0) model.

## Supporting Information

Figure S1 Simulations from Our Neutral Model The blue lines depict the 25% and 75% quantiles of the allelic skew, and the red line depicts the median skew, as a function of mutation frequency.
(6 KB PDF)

Figure S2 Alternate Testes Dissection Strategy Testis 854–1. The testis was divided in half as in Figure 1 and then the head half was cut in half again parallel to the epididymal axis to produce two pieces containing ∼1/4–1/3 of the whole testis. The portion labeled UL (i) was then divided into 192 pieces each approximately four times smaller than was the case for testis 374. The UR (ii) and the TH (iii) portions were dissected so that the average piece size was approximately the same as that obtained for testis 374.
(2.1 MB PDF)

Figure S3 Annealing of Mutant-Specific PAP Primers to Wild-Type and Mutant DNA Templates For additional information, see Methods. Only the 3′ portions of the primer sequences are shown.
(12 KB PDF)

Figure S4 Example of PAP Results Ten PAP reactions were carried out on DNA from a piece of Testis 854–2.
(A) Amplification curves (linear plot) show the increase in fluorescence as a function of cycle number. Note that the displacement of the curves from one another does not signal different starting amounts of target DNA, because on average, 90% of the wells should have no more than one target according to the Poisson distribution. Five of the samples gave positive curves and five were negative.
(B) Melting profiles of the ten reactions shown in (A). Only the five positive reactions gave the correct melting profile for the expected PAP product. The negative control reactions (unpublished data) indicated no product was formed.
(75 KB PDF)

Protocol S1 Computer Simulation Program (55 KB DOC)

Table S1 C755G Mutation Frequency and Genome Number (Testes 374 and 854) (52 KB XLS)

Table S2 Control Mutation Frequency and Genome Number (Testis 374) (40 KB XLS)

Text S1 Supporting Information (22 KB PDF)

### Accession Numbers

The Online Mendelian Inheritance in Man (www.ncbi.nlm.nih.gov/omim/) accession number for Apert syndrome is 101200. The National Center for Biotechnology Information (NCBI; http://www.ncbi.nlm.nih.gov/) Nucleotide accession number for the *FGFR2* gene is NM_000141. The Single Nucleotide Polymorphism database (http://www.ncbi.nlm.nih.gov/projects/SNP/) build 127 transversion for the C nucleotide site on human Chromosome 7 is rs3801993.

## Abbreviations

CpG: dinucleotide containing a C followed by a G (5′ to 3′)
FGFR2: fibroblast growth factor receptor 2
FGFR3: fibroblast growth factor receptor 3
PAP: pyrophosphorolysis-activated PCR
SrAp: self-renewing Ap spermatogonia
