# Supplementary material for: The Molecular Anatomy of Spontaneous Germline Mutations in Human Testes
Source: PLoS Biol. 2007 Aug 28;5(9):e224. doi: 10.1371/journal.pbio.0050224 (PMC1951783; doi:10.1371/journal.pbio.0050224)
Supplement: Supplementary file 1 [file pbio.0050224.sd002.pdf]

Supporting Information , Qin et al.

Text S1) Sperm data require positive selection?

In [1], the researchers considered two mutations (C>G and C>T) at the 755 disease site. They exploited the fact that near this site there is a relatively high frequency SNP. For individuals who were heterozygous at this SNP, the researchers measured the mutation frequency for the C>G and C>T mutations separately and the proportion of each mutant type that carried one specific allele at the nearby SNP. They repeated these measurements for ~50 informative individuals. Then for each mutation the researchers computed the average mutation frequency and the SNP allelic skew across this population sample. The intuition was that if many *de novo* mutations at nucleotide 755 had occurred then in each individual the SNP proportion value for the specified allele would be near 50% so the skew would be low, whereas if a small number of *de novo* mutations had occurred then in each individual the proportion value for the SNP allele would be nearer to 0% or 100% and the skew would be high. They found that the C>G mutation had both a higher average frequency and a greater allelic skew than the C>T mutation. They argued that no reasonable neutral model could explain why the mutation with the higher mutation frequency could also have the higher SNP allele skew and concluded that the germline cells harboring the C>G mutation must undergo positive selection.

Figure S1 questions this conclusion. We simulated the neutral model discussed in this paper (p=0, 30 growth phase generations, 1,000 adult phase generations, mutation rates per cell division ranging from 10^{-9} to 10^{-7}, see Methods in paper for details). For a given mutation rate, we simulated the mutation frequencies and SNP allele proportion values for 50 individuals. For this hypothetical sample, we calculated the average mutation frequency and the SNP allelic skew. (As in the Supplemental online materials of [1], this allelic skew is defined as the variance of the proportion

values across the population sample.) For the given mutation rate, we repeated the process 1,000 times to get a distribution for the allelic skew values. We then repeated the entire process for a range of mutation rates. Figure S1 shows the 25% and 75% quantiles of the allelic skew in blue and the median skew in red as a function of mutation frequency.

In [1], the researchers argued that for any reasonable neutral model the SNP allelic skew would decrease with frequency. This appears to be true for the median skew values, but the process is very noisy. For two frequencies, representing the two different 755 nucleotide mutations (C>G and C>T), there is a substantial probability that the sample with the higher frequency would also have a higher SNP allelic skew. Thus in our opinion, the evidence in [1] does not require a selection explanation. In order for the observation that the mutation with a higher frequency also has a higher skew to mandate such an explanation, we would expect the predictions of the neutral model to be less noisy; the median and quantile values would decrease such that for two different frequencies the SNP allelic skew distributions would overlap only with a small probability. As can be seen from Figure S1, the overlap is considerable for this model.

Testes DNA purification and quantitation

DNA was extracted from all testis pieces using a Puregene kit (Gentra Systems). The concentrations of all DNA samples were determined by real-time PCR on an Opticon 2 instrument (Bio-Rad, Hercules, CA) using human genomic DNA (Clontech, Mountain View, CA) as standard. The real-time PCR assays were performed in 50-µl volumes containing 1× Gold PCR buffer, 2 mM $MgCl_2$, 80 µM dNTPs, 0.16 µM primers (5' GGGTGTTTCTCTTGGCTTCC and 5' TGCTAAGCCCCACATTATCC amplifying a 178 bp genomic region on chromosome 21:40235566-40235743), 2uM Rox, 0.2X Sybr Green I, 1 unit *AmpliTaq* Gold DNA Polymerase (Applied Biosystems, Foster City, CA).

The cycling conditions included an initial denaturation step of 10 minutes at 94°C and 50 PCR cycles. Each cycle consisted of 30 seconds at 94°C and 1 minute at 63°C. The cycling was followed by analysis of the melting curves. The amount of DNA in each reaction was determined by comparison with a standard curve generated with 100,000, 10,000, 1,000, and 100 human genomes. All samples were amplified in duplicate. The numbers of genomes for each testis piece are presented in Table S1.

On average, each testis piece (except the 192 pieces from the ¼ portion of testis 854-1) contained DNA from about $1.6x10^7$ cells. This estimate was made using the average number of genomes per testis piece and data on the number of germline and non-germline cells found in individual human testes [2] after accounting for the ploidy of pre- and post-meiotic germline cells as well as non-germline cells (Sertoli cells). Variation in the amount of DNA among the pieces appears to be a function of their size which is not uniform since it is influenced ultimately by the oval shape of the testis (See Figure 1 and Figure S2).

References

1. Goriely A, McVean GA, Rojmyr M, Ingemarsson B, Wilkie AO (2003) Evidence for selective advantage of pathogenic FGFR2 mutations in the male germ line. Science 301: 643-646.
2. Zhengwei Y, Wreford NG, Royce P, de Kretser DM, McLachlan RI (1998) Stereological evaluation of human spermatogenesis after suppression by testosterone treatment: heterogeneous pattern of spermatogenic impairment. J Clin Endocrinol Metab 83: 1284-1291.
