# Supplementary material for: The Molecular Anatomy of Spontaneous Germline Mutations in Human Testes
Source: PLoS Biol. 2007 Aug 28;5(9):e224. doi: 10.1371/journal.pbio.0050224 (PMC1951783; doi:10.1371/journal.pbio.0050224)
Supplement: Supplementary file 2 [file pbio.0050224.sg001.pdf]

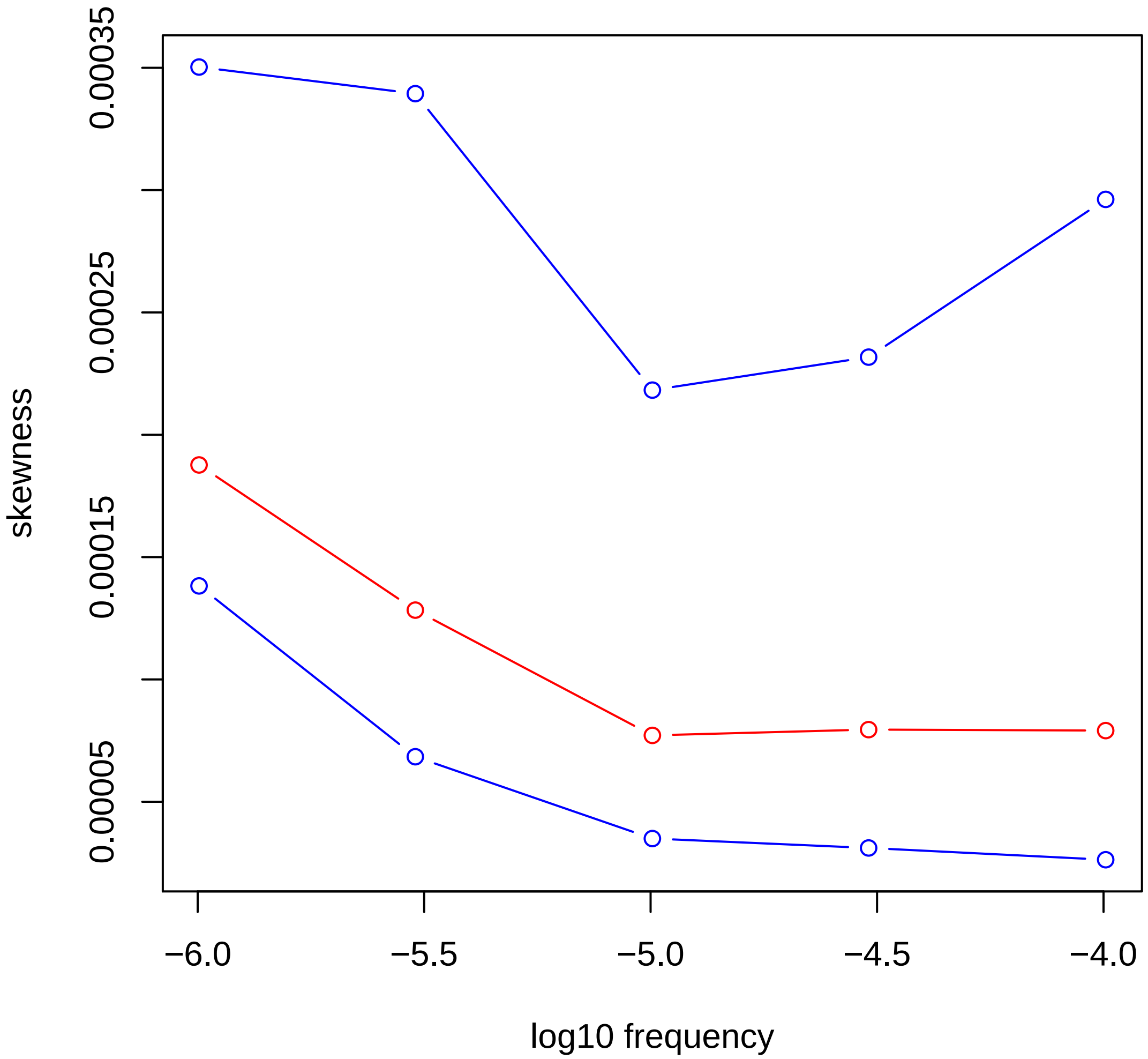

0.00035
0.00025
0.00015
0.00005
skewness
−6.0
−5.5
−5.0
−4.5
−4.0
log10 frequency
