# Supplementary material for: The Molecular Anatomy of Spontaneous Germline Mutations in Human Testes
Source: PLoS Biol. 2007 Aug 28;5(9):e224. doi: 10.1371/journal.pbio.0050224 (PMC1951783; doi:10.1371/journal.pbio.0050224)
Supplement: Supplementary file 3 [file pbio.0050224.sg002.pdf]

FIGURE S2

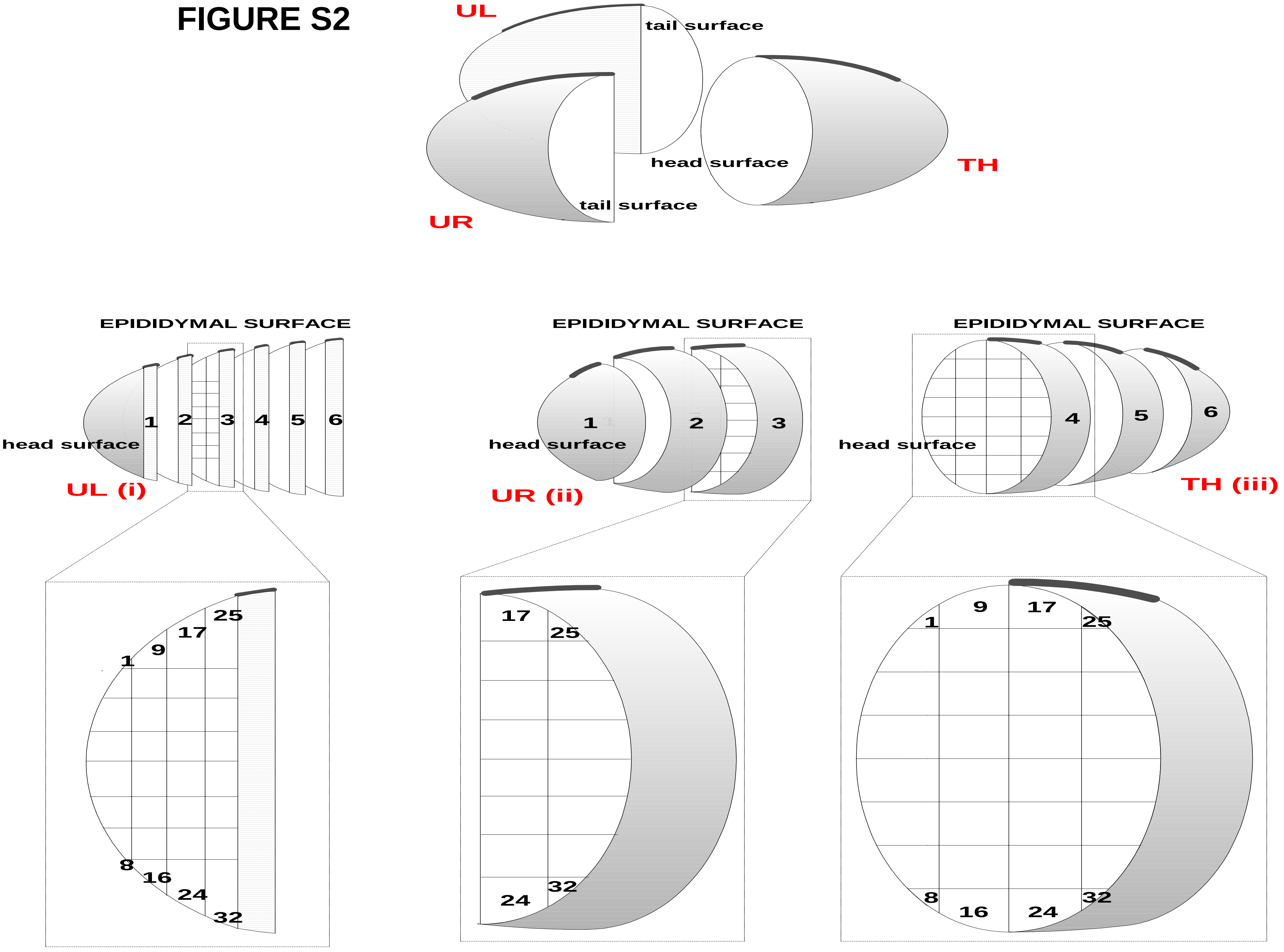

UL
tail surface
head surface
TH
tail surface
UR
EPIDIDYMAL SURFACE
1
2
3
4
5
6
head surface
UL (i)
EPIDIDYMAL SURFACE
1
2
3
head surface
UR (ii)
EPIDIDYMAL SURFACE
4
5
6
head surface
TH (iii)
25
17
9
1
8
16
24
32
17
25
32
24
1
9
17
25
8
16
24
32
