# Supplementary material for: The Molecular Anatomy of Spontaneous Germline Mutations in Human Testes
Source: PLoS Biol. 2007 Aug 28;5(9):e224. doi: 10.1371/journal.pbio.0050224 (PMC1951783; doi:10.1371/journal.pbio.0050224)
Supplement: Supplementary file 4 [file pbio.0050224.sg003.pdf]

Figure S3

C755G APERT MUTANT TEMPLATE

5′---tctccaccagagcgatGgcctcaccggcccatc---

ddCcggagtggccgggtag---

---tctccaccagagcgatGdd

---agaggtggtctcgctaCcggagtggccgggtag---5′

WILD TYPE TEMPLATE

5′---TCTCCACCAGAGCGATCGCCTCACCGGCCCATC---

ddC CGGAGTGGCCGGGTAG---

Gdd

---TCTCCACCAGAGCGAT

---AGAGGTGGTCTCGCTAGCGGAGTGGCCGGGTAG---5′
