# Supplementary material for: The Molecular Anatomy of Spontaneous Germline Mutations in Human Testes
Source: PLoS Biol. 2007 Aug 28;5(9):e224. doi: 10.1371/journal.pbio.0050224 (PMC1951783; doi:10.1371/journal.pbio.0050224)
Supplement: Supplementary file 5 [file pbio.0050224.sg004.pdf]

Figure S4

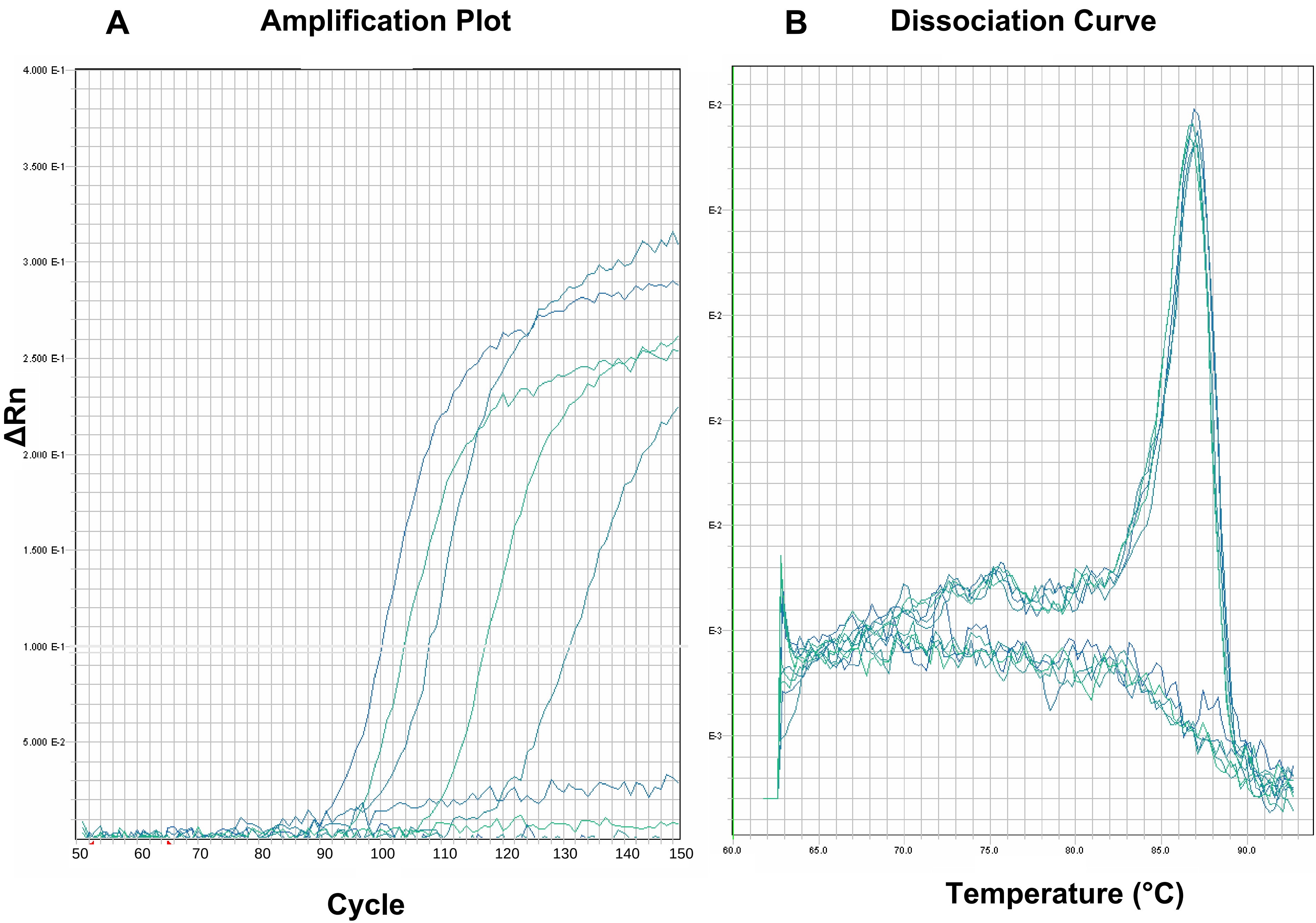

A
Amplification Plot
ΔRn
4.000 E-1
3.500 E-1
3.000 E-1
2.500 E-1
2.000 E-1
1.500 E-1
1.000 E-1
5.000 E-2
50
60
70
80
90
100
110
120
130
140
150
Cycle
B
Dissociation Curve
E-2
E-2
E-2
E-2
E-2
E-3
E-3
60.0
65.0
70.0
75.0
80.0
85.0
90.0
Temperature (°C)
