# Supplementary material for: The Molecular Anatomy of Spontaneous Germline Mutations in Human Testes
Source: PLoS Biol. 2007 Aug 28;5(9):e224. doi: 10.1371/journal.pbio.0050224 (PMC1951783; doi:10.1371/journal.pbio.0050224)
Supplement: Supplementary file 6 [file pbio.0050224.sd001.doc]

The computer code to simulate the hotspot (p=0) and selection (p>0) models is included below. The code can also be freely downloaded from the website: “<http://www.cmb.usc.edu/people/petercal/>”. The code is written in C++ and needs to be compiled. On a typical UNIX system, the compile command is “g++ -o tsimm tsimm.cpp”. An example execution command is then,

./tsimm 1234 27 1127 0 1 100 10 .00000029 .00000001 .00038 .00019 .05 0

Here is an explanation for the parameters:

1. 1234 is the random number seed: any integer will do.
2. 27 is the number of growth phase generations: in the paper we consider 27 and 34.
3. 1127 is the number of adult phase generations: in the paper we consider 1127 and 934.
4. 0 is p, the probability a mutant in the adult phase divides symmetrically: for the hotspot model we set p=0, for the selection model we optimize over p>0.
5. 1 is the factor of decrease in the number of cells during the adult phase: in the paper we fix this parameter at 1.
6. 100 is the number of repetitions.
7. 10 is the number of lambda values (mutation rate per cell division).
8. .00000029 is the first lambda value considered.
9. .00000001 is the increment in lambda values.
10. .00038 is the target total testis mutation frequency.
11. .000019 is the allowed tolerance for the total testis mutation frequency (so any frequency between .00038 +/- .000019 is accepted).
12. .05 is the target for the minimum fraction of testis pieces which contain at least 95% of the mutant cells.
13. 0 is the allowed tolerance for the above quantity.

There are several cases for the output:

1. If the number of repetitions equals 1, then the output is the mutation frequencies of the 192 testis pieces.
2. If the number of repetitions is greater than 1, and the number of lambda values equals 1, then the output is the total mutation frequency and the minimum fraction of testis pieces which contain at least 95% of the mutants.
3. If the number of repetitions is greater than 1, and the number of lambda values is greater than 1, then the output is for each lambda value: the lambda value, the p value, and the acceptance probability. If the last parameter is 0, a simulation is accepted based solely on the total testis mutation frequency; if the last parameter is greater than 0, a simulation is accepted based both on the total testis mutation frequency and the minimum fraction of testis pieces which contain at least 95% of the mutants.

```
#include <vector>
#include <iostream>
#include <fstream>
#include <iomanip>
#include <math.h>
using namespace std;

double normal(double,double);
int poisson(double);
double myrand(double,double);
void space(double [6][8][4],double,double);
double grow(double,int,double,double);
double getp95(double [6][8][4]);

main(int argc, char *argv[]) {

  int growgens,adultgens,numlambda,nreps,i,j,ii,jj,kk,succ,itime,seed;
  double p,q,lambda,lowlambda,inclambda,tarfreq,errfreq,tarp95,errp95,extra,qq,freq,p95,mtot,dtemp,muts,x,tot;
  double testis[6][8][4];

  // INPUT PARAMETERS
  if (argc < 9) {
    cerr << "Too few input parameters" << endl;
    exit(1);
  }
  else {
    seed = atoi(argv[1]);      // RANDOM NUMBER SEED
    growgens = atoi(argv[2]);  // NUMBER GROWTH PHASE GENERATIONS
    adultgens = atoi(argv[3]); // NUMBER ADULT PHASE GENERATIONS
    p = atof(argv[4]);         // IN THE ADULT PHASE: PROBABILITY A MUTANT DIVIDES SYMMETRICALLY
    q = atof(argv[5]);         // FACTOR OF DECREASE IN NUMBER OF CELLS OVER ALL ADULT PHASE GENERATIONS: 1 MEANS NO CHANGE. 2 MEANS HALF GONE BY END.
    nreps = atoi(argv[6]);     // NUMBER REPS
    numlambda = atoi(argv[7]); // NUMBER OF DIFFERENT LAMBDA VALUES (MUTATION RATES) TO CONSIDER
    lowlambda = atof(argv[8]); // FIRST (LOWEST) LAMBDA VALUE
    if (argc > 9) {
      inclambda = atof(argv[9]); // LAMBDA INCREMENT
      tarfreq = atof(argv[10]);  // TARGET TOTAL TESTIS MUTATION FREQUENCY
      errfreq = atof(argv[11]);  // ALLOWED ERROR IN ABOVE
      tarp95 = atof(argv[12]);    // TARGET PERCENT OF TESTIS PIECES NECESSARY TO COMPRISE 95% OF MUTANTS
      errp95 = atof(argv[13]);    // ALLOWED ERROR IN ABOVE
    }
    else { inclambda = 0; }
  }

  // SET-UP
  srand48(seed);
  if (q > 1) {
    qq = 1-pow(q,(-1/((double)(adultgens)))); // IN THE ADULT PHASE: PROBABILITY A CELL (MUTANT OR NOT) DIES AND IS NOT REPLACED
    mtot = round(pow(2,((double)(growgens)))/q);
  }
  else {
    qq = 0;
    mtot = pow(2,((double)(growgens)));
  }
  lambda = lowlambda - inclambda;

  for (i=0;i<numlambda;i++) {
    lambda += inclambda;
    succ = 0;
    for (j=0;j<nreps;j++) {

      // FORMAT
      extra = 0;
      for (ii=0;ii<6;ii++) {
	for (jj=0;jj<8;jj++) {
	  for (kk=0;kk<4;kk++) {
	    testis[ii][jj][kk] = 0.;
	  }
	}
      }

      // GROWTH PHASE
      for (ii=0;ii<growgens;ii++) {
	dtemp = pow(2,((double)(ii+1)));
	muts = myrand(dtemp,lambda);
	if (muts > 0) {
	  dtemp = pow(2,((double)(growgens-ii-1)));
	  for (jj=0;jj<muts;jj++) {
	    itime = adultgens;
	    x = grow(dtemp,itime,p,qq);
	    extra += x - (dtemp/q);
	    space(testis,x,mtot);
	  }
	}
      }

      // ADULT PHASE
      tot = pow(2,((double)(growgens)));
      for (ii=0;ii<adultgens;ii++) {
	tot = round(tot*(1-qq));
	muts = myrand(tot,lambda);
	if (muts > 0) {
	  itime = adultgens - ii;
	  for (jj=0;jj<muts;jj++) {
	    x = grow(1.,itime,p,qq);
	    extra += x - pow((1-qq),((double)(itime)));
	    space(testis,x,mtot);
	  }
	}
      }

      // OUTPUT
      mtot += ((int)(round(extra)));

      if (nreps == 1) { //THIS CASE OUTPUTS MUTATION FREQUENCIES FOR 192 PIECES
	for (ii=0;ii<6;ii++) {
	  for (jj=0;jj<8;jj++) {
	    for (kk=0;kk<4;kk++) {
	       cout << testis[ii][jj][kk]/mtot << endl;
	    }
	  }
	}
      }
      else {
	freq = 0;
	for (ii=0;ii<6;ii++) {
	  for (jj=0;jj<8;jj++) {
	    for (kk=0;kk<4;kk++) {
	      freq += testis[ii][jj][kk];
	    }
	  }
	}
	freq = freq/mtot;
	p95 = getp95(testis);
	if (errp95 > 0) {
	  if (((tarfreq-errfreq) < freq) && (freq < (tarfreq+errfreq)) && ((tarp95-errp95) < p95) && (p95 < (tarp95+errp95))) { succ += 1; }
	}
	else {
	  if (((tarfreq-errfreq) < freq) && (freq < (tarfreq+errfreq))) {
	    succ += 1;
	  }
	}
	if (numlambda == 1) { //THIS CASE OUTPUTS FREQ AND p95 VALUES
	  cout << freq << " " << p95 << endl;
	}
      }
    }

    // THIS CASE OUTPUTS THE LIKELIHOOD FOR MANY LAMBDA VALUES
    if (numlambda > 1) {
	cout << lambda << " " << p << " " << ((double)(succ))/((double)(nreps)) << endl;
    }
  }

  return 1;
}

double getp95(double testis[6][8][4]) {
  // OUTPUT THE PERCENTAGE OF TESTIS PIECES REQUIRED TO COMPRISE 95% OF THE MUTANTS
  // INPUT 3-DIMENSIONAL TESTIS ARRAY

  int i,j,k,cnt,ibig;
  double big,tot,lin[192];

  cnt = -1;
  for (i=0;i<6;i++) {
    for (j=0;j<8;j++) {
      for (k=0;k<4;k++) {
	cnt += 1;
	lin[cnt] = testis[i][j][k];
	tot += testis[i][j][k];
      }
    }
  }
  for (i=0;i<191;i++) {
    big = lin[i];
    for (j=(i+1);j<192;j++) {
      if (lin[j] > big) {
	big = lin[j];
	ibig = j;
      }
    }
    if (big > lin[i]) {
      lin[ibig] = lin[i];
      lin[i] = big;
    }
  }

  cnt = -1;
  big = 0.;
  while ((big/tot) < 0.95) {
    cnt += 1; if (cnt > 192) { cerr << "Problem in getp95." << endl; exit(1); }
    big += lin[cnt];
  }

  return ((double)(cnt))/192;
}

double grow(double sta,int itime,double p,double qq) {
// OUTPUT NUMBER OF MUTANTS IN THE CLUSTER
// INPUT INITIAL NUMBER MUTANTS (1 FOR ADULT PHASE, MORE FOR GROWTH PHASE),
// TIME REMAINING IN GENERATIONS, p AND qq PARAMETERS DISCUSSED ELSEWHERE

  int i;
  double x;
  x = sta;
  if (p > 0) {
    if (qq > 0) {
      for (i=0;i<itime;i++) {
	x += myrand(x,p);
	x -= myrand(x,qq);
	if (x <= 0) {
	  x = 0;
	  break;
	}
      }
    }
    else {
      for (i=0;i<itime;i++) {
	x += myrand(x,p);
      }
    }
  }
  else {
    if (qq > 0) {
      for (i=0;i<itime;i++) {
	x -= myrand(x,qq);
	if (x <= 0) {
	  x = 0;
	  break;
	}
      }
    }
  }

  return x;
}

void space(double testis[6][8][4],double mclump,double mtot) {
  // OUTPUT DISTRIBUTES MUTATION CLUSTER INTO 3-DIMENSIONAL TESTIS ARRAY
  // INPUT 3-DIMENSIONAL TESTIS ARRAY,
  // NUMBER OF MUTANTS IN CLUMP, TOTAL NUMBER OF CELLS IN TESTIS

  double x,y,z,x1,x2,y1,y2,z1,z2,leng,dtemp;
  int i,j,k;

  x = 6.*drand48();
  y = 8.*drand48();
  z = 4.*drand48();
  leng = pow( 192.*mclump/mtot, 0.333);

  if (leng > 4) {
    dtemp = round(mclump/192.);
    for (i=0;i<6;i++) {
      for (j=0;j<8;j++) {
	for (k=0;k<4;k++) {
	  testis[i][j][k] += dtemp;
	}
      }
    }
  }
  else {
    if (x-(0.5*leng) < 0) { x += (0.5*leng)-x; }
    if (x+(0.5*leng) > 6) { x -= x+(0.5*leng)-6; }
    if (y-(0.5*leng) < 0) { y += (0.5*leng)-y; }
    if (y+(0.5*leng) > 8) { y -= y+(0.5*leng)-8; }
    if (z-(0.5*leng) < 0) { z += (0.5*leng)-z; }
    if (z+(0.5*leng) > 4) { z -= z+(0.5*leng)-4; }
    for (i=0;i<6;i++) {
      for (j=0;j<8;j++) {
	for (k=0;k<4;k++) {
	  x1 = max( ((double)(i)),  (x-(0.5*leng)) );
	  x2 = min( ((double)(i+1)),(x+(0.5*leng)) );
	  y1 = max( ((double)(j)),  (y-(0.5*leng)) );
	  y2 = min( ((double)(j+1)),(y+(0.5*leng)) );
	  z1 = max( ((double)(k)),  (z-(0.5*leng)) );
	  z2 = min( ((double)(k+1)),(z+(0.5*leng)) );
	  if ((x2 > x1) && (y2 > y1) && (z2 > z1)) {
	    dtemp = (x2-x1)*(y2-y1)*(z2-z1);
	    testis[i][j][k] += round( mclump*dtemp/pow(leng,3.) );
	    if (testis[i][j][k] > (mtot/192.) ) {
  	      cerr << “Problem in space” << endl;
	      //exit(1);
    }
	  }
	}
      }
    }
  }
}

double normal(double mean,double sd) {
  double y1,y2,x;
  int flag;
  flag = 1;
  while (flag) {
    y1 = -log(drand48());
    y2 = -log(drand48());
    if (y2 >= (.5*(y1-1)*(y1-1))) {
      if (drand48() < .5) { x = y1; }
      else { x = -y1; }
      flag = 0;
    }
  }
  x = mean + x*sd;
  return x;
}

int poisson(double x) {
  double temp;
  int flag, cnt;
  temp = 0;
  flag = 1;
  cnt = 0;
  while (flag) {
    cnt += 1;
    temp = temp + log(drand48());
    if (temp < (-x)) {
      flag = 0;
      cnt -= 1;
    }
  }
  return cnt;
}

double myrand(double num,double q) {
// POISSON. IF NUM*Q HIGH THEN NORMAL APPROXIMATION TO SPEED UP THE ALGORITHM

  double dnum,temp;

  if (num > 0) {
	dnum = num*q;
	if (dnum < 20) {
	  temp = ((double)(poisson(dnum)));
	}
	else {
	  temp = round(normal(dnum,(sqrt(dnum))));
	}
	if (temp < 0) { temp = 0.; }
  }
  else { temp = 0.; }
  return temp;
}
```
